# Cell Type-Specific Adhesion and Migration on Laser-Structured Opaque Surfaces

**DOI:** 10.3390/ijms21228442

**Published:** 2020-11-10

**Authors:** Jörn Schaeske, Elena Fadeeva, Sabrina Schlie-Wolter, Andrea Deiwick, Boris N. Chichkov, Alexandra Ingendoh-Tsakmakidis, Meike Stiesch, Andreas Winkel

**Affiliations:** 1 Department of Prosthetic Dentistry and Biomedical Materials Science, Hannover Medical School, Carl-Neuberg-Straße 1, 30625 Hannover, Germany; Joern.Schaeske@gmx.de (J.S.); ingendoh-tsakmakidis.alexandra@mh-hannover.de (A.I.-T.); Stiesch.Meike@mh-hannover.de (M.S.); 2 Institute of Quantum Optics, Leibniz University of Hannover, Welfengarten 1, 30167 Hannover, Germany; elena.fadeeva@gmx.de (E.F.); schlie-wolter@skc-beratung.de (S.S.-W.); deiwick@iqo.uni-hannover.de (A.D.); chichkov@iqo.uni-hannover.de (B.N.C.)

**Keywords:** biomaterials, cytocompatibility, focal adhesion, cell exclusion assay, spike structures, in vitro screening, primary vs. immortalized cell lines, cell proliferation, cell spreading

## Abstract

Cytocompatibility is essential for implant approval. However, initial in vitro screenings mainly include the quantity of adherent immortalized cells and cytotoxicity. Other vital parameters, such as cell migration and an in-depth understanding of the interaction between native tissue cells and implant surfaces, are rarely considered. We investigated different laser-fabricated spike structures using primary and immortalized cell lines of fibroblasts and osteoblasts and included quantification of the cell area, aspect ratio, and focal adhesions. Furthermore, we examined the three-dimensional cell interactions with spike topographies and developed a tailored migration assay for long-term monitoring on opaque materials. While fibroblasts and osteoblasts on small spikes retained their normal morphology, cells on medium and large spikes sank into the structures, affecting the composition of the cytoskeleton and thereby changing cell shape. Up to 14 days, migration appeared stronger on small spikes, probably as a consequence of adequate focal adhesion formation and an intact cytoskeleton, whereas human primary cells revealed differences in comparison to immortalized cell lines. The use of primary cells, analysis of the cell–implant structure interaction as well as cell migration might strengthen the evaluation of cytocompatibility and thereby improve the validity regarding the putative in vivo performance of implant material.

## 1. Introduction

Bone-anchored implant systems, which are widely used in modern dentistry and orthopedics, are based on good tissue integration to compensate for lost functions of natural tissue [1,2]. Accordingly, after decades of development and improvement, the products on the market, mainly made of titanium, titanium alloys, and ceramics, are characterized by a good healing rate, high load capacity, and adequate force transmission [3]. Nevertheless, there is still a need for further optimization to avoid certain problems during implant treatment [4,5,6]. Besides implant-associated infections, deficient integration into the surrounding tissue presents a major complication [7]. Over the years, researchers have pursued various approaches to optimize implant materials and especially their surfaces by innovative coatings and structuring, aiming at improved properties regarding antibacterial efficacy, accelerated wound healing, or forced tissue formation [8,9,10,11]. Despite frequently published promising results according to in vitro and initial preclinical studies, only a few of these approaches have actually been implemented in medical products and a groundbreaking relevance of these innovations for patients cannot yet be confirmed [12].

As part of an initial screening to test the suitability of new implant strategies, in vitro studies provide the important first indications for a putative reaction of relevant cell types in the peri-implant tissue and usually address questions related to the approval process. Particularly, they focus on their compatibility/toxicity or the basic functionality, especially the influence on the initial adhesion and its quantification (e.g., EN ISO 10993-5 Biological evaluation of medical devices—Part 5: Testing for in vitro cytotoxicity; EN ISO 7405 Assessment of the biocompatibility of medical devices in dentistry) [13]. Immortalized cell lines (even of xenogenic origin) are used in the majority of cases, as they are easy to isolate/cultivate and, due to their homogeneity, have a high reproducibility of results [14,15]. Compared to in vivo studies, they are ethically less problematic, faster, cheaper, and better to standardize and reproduce, which explains their high relevance, especially for the initial screening of novel materials and surfaces [16].

Contrary to expectations, large differences can occur when in vitro results based on immortalized cell lines are directly transferred to the performance observed in clinical application [17,18]. One reason is that the interaction of individual cells from a tissue under rudimentary in vitro culture conditions cannot be equated with the complexity of a tissue network with the interaction of the most diverse cell systems, extracellular messengers, and a constantly changing supply of nutrients and oxygen during injury or regeneration [19,20]. This difference is intensified by the use of highly proliferative cell systems with low variability in behavior, as this is in contrast to the individuality of the patients and the heterogeneity of cells in the differentiated target tissues [21,22]. Furthermore, other parameters play an important role in the functional interaction of human cells with artificial materials, surfaces, and structures, which can provide additional information in advance about the expected clinical performance [23]. The qualitative evaluation of adhesion and three-dimensional observation of the interaction with the surface, as well as the proliferation, migration, and differentiation of cells, are underrepresented in most screenings to date [24,25,26,27,28,29]. This is partly due to the fact that corresponding investigation methodology cannot yet be technically implemented for all surfaces or are too costly for a broad application to a large number of surface modifications. For example, the classical wound healing assay (scratch assay) for investigation of the migration behavior of adherent cells on structured or coated surfaces cannot be performed without causing damage. Boundary or cell exclusion assays would be an alternative, but have so far only been performed using light microscopy on transparent surfaces [30]. Additionally, most migration studies are limited to a few days, which slightly reflect the lengthy healing process in vivo [31,32].

Micro- and nanostructured surfaces have an advantage; the purely physical modification of the surface does not change the underlying chemical properties of the material (e.g., its cytotoxicity). Nevertheless, these modified structures can have a massive effect on cell growth and subsequently, on the material’s integration into the target tissue [7,33,34]. Ultra-short pulse laser ablation is a special processing method in which a laser beam with a defined energy and frequency removes material in a single step. The method can be performed on many materials, is flexible, and enables the precise production of a large number of complex geometries in the micro- and nanometer range [35,36]. Although topographies resulting from this method have not yet been applied to commercial implants, they have demonstrated to allow the selective growth of specific cell types in certain implant areas (e.g., to avoid connective tissue capsules during bone healing) [37,38,39]. This altered cell behavior in vitro also makes these modifications interesting for the further development of test methods with regard to a greater significance for expected performance in patients.

The aim of this study was to investigate in greater detail than in standard biomaterial screening, the adhesion, proliferation, and migration of tissue cells on structured titanium surfaces. Therefore, in vitro screenings were optimized and combined with fluorescence as well as confocal laser scanning microscopy and a new adequate model had to be developed to study the migration of cells on non-transparent surfaces. Besides the different surface structures, cells from different tissues (fibroblasts and osteoblasts) were also used as primary cells or as an immortalized murine version to evaluate our extended in vitro screening.

## 2. Results

### 2.1. Defined Spike Spacing on Titanium-Coated Silicon Plates

**Figure 1:**
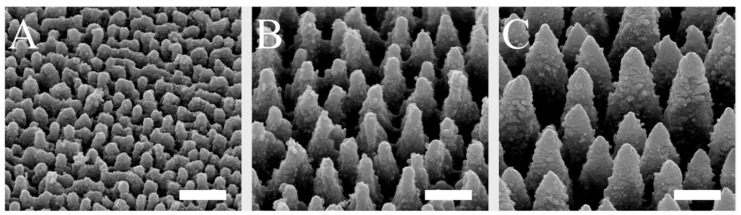
SEM images of small (**A**), medium (**B**), and large spikes (**C**). These spike structures were produced on silicon plates by laser ablation and then coated with titanium. Scale = 5 µm.

The spike spacing was selected in a range that has been shown to affect cell behavior on implant surfaces [29,38,40]. The spike structures, which were homogeneously produced on silicon plates by laser ablation and coated with titanium, could be created in a highly reproducible and uniform manner over the surface and could be visualized by scanning electron microscopy (Figure 1). By using different laser pulse energies, spikes with different dimensions and distances to each other were generated. The spike topographies were successfully produced according to the required spike spacing, with an average distance of 2.3 µm (small spikes; S), 5.5 µm (medium spikes, M), or 7.6 µm (large spikes; L).

### 2.2. Spike Distance Influences Cell Adhesion and Proliferation of Peri-Implant Tissue Cells

**Figure 2:**
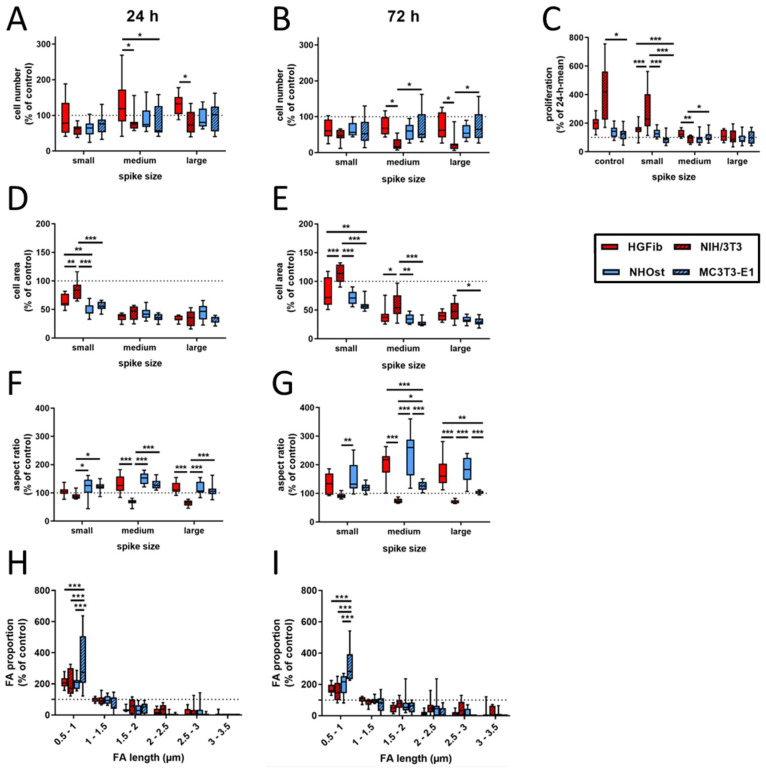
Cell type-specific reactions on spike topographies (direct comparison of the different cell type performances; for direct comparison of the different spike size effects, see Supplementary Figure S3). HGFib, NIH/3T3, NHOst, and MC3T3-E1 were cultivated for 24 or 72 h on the spike structures. The cell number was determined by DAPI staining (**A**,**B**); the cell area (**D**,**E**) and aspect ratio (**F**,**G**) were determined by phalloidin-TRITC staining. FA length was measurable only on the small spikes. The staining was done by anti-pFAK and DyLight488 (**H**,**I**). For calculation of cell proliferation, cell numbers after 72 h were normalized to the corresponding 24 h mean value (**C**). Data are visualized by boxplots of nine replicates of at least three independent experiments. Statistics were performed by two-way-ANOVA (* *p* < 0.05, ** *p* < 0.01, *** *p* < 0.001).

In contrast to an exclusive quantification of adherent cells at different time points, additional information for cell area, aspect ratio, focal adhesion length, and composition were collected in the present study, which allow a more detailed picture of the quality of this attachment on the different surfaces using various cell types. Figure 2 represents the cell type-specific reactions on spike topographies focused on the direct comparison of the different cell type performances. Corresponding exemplary fluorescence images of adhered cells on different surfaces after 24 and 72 h as well as graphs focusing on the direct comparison of the different spike size effects are provided in Supplementary Figures S1–S3. The average number of adherent cells was reduced on all structures compared to the control after 24 h, with the exception of primary fibroblasts. For NIH/3T3, NHOst, and MC3T3-E1 it seemed to be of little relevance which dimensions the structure had. Only HGFib showed a preference for medium-sized and especially large structures, on which more cells adhered on average than on unstructured surfaces but without achieving significance compared to the control (Figure 2A). After 72 h, all investigated cell populations showed on all structures a lower cell count than on the control areas. A specific influence of any topography on the deposition behavior was in no case evident at this time point (Figure 2B). However, when comparing the different cell populations, the cell numbers for NIH/3T3 on the medium and large structures were significantly lower compared to HGFib and MC3T3-E1, which highlights the negative influence of these topographies, especially on the adhesion of this cell type (Figure 2B).

By comparing both time points, the proliferation behavior of the individual cell populations on the different surfaces can be deduced. Proliferation was consistently the strongest for all cell populations on the unstructured control surfaces, usually followed by growth on small spikes with the exception for MC3T3-E1, where large spikes had less negative effects (Figure 2C). The immortalized NIH/3T3 were significantly more proliferative than primary fibroblasts. Little growth, if not stagnation or reduction, in adherent cells on medium and large spikes was observed during investigation. Osteoblasts barely proliferated within 2 days on the investigated surfaces, and it seems not relevant whether cells are primary human or immortalized murine osteoblasts.

**Figure 3:**
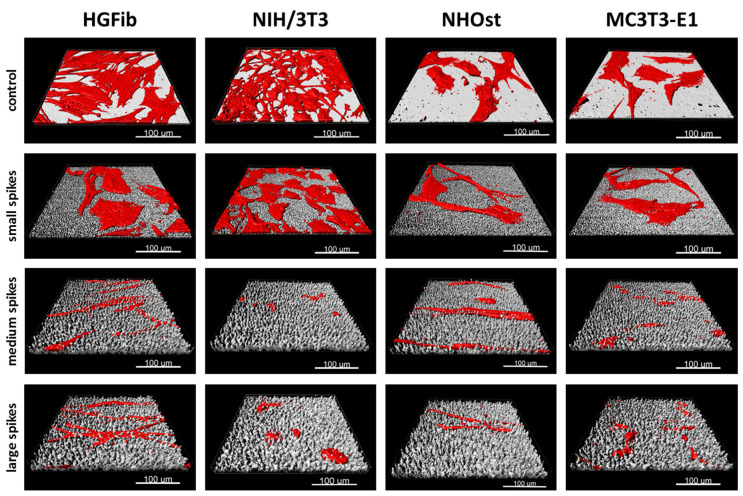
Exemplary 3D reconstructions of HGFib, NIH/3T3, NHOst, and MC3T3-E1 after 72 h culture on flat control surfaces, small spikes, medium spikes, and large spikes. The cell reconstructions were based on the actin filament staining with phalloidin-TRITC and the surface topography was visualized using light reflection at 638 nm.

The morphological changes of the adherent cells on spike structures compared to the unstructured control area could be determined by two parameters, the cell area and cell shape (aspect ratio). Regarding the cell area, it was found that there was a reduction in size on the spike structures, except for the NIH/3T3 on the small structures after 72 h (Figure 2D,E and Figure 3). The reduction in the cell area was particularly pronounced on the medium-sized and large structures. This turned out to be highly significant compared to the small structures after one day of attachment, except for primary osteoblasts. The cell shape of primary cells was dependent on spike size (Figure 2F,G). This dependence was more pronounced after 72 h than after 24 h. Both HGFib and NHOst were significantly longer on the medium spikes than on the small and large spikes (Figure 3). From the immortalized cells, only the NIH/3T3 were significantly longer on the small spikes than on the large spikes after 24 h but were less influenced by the presented spike distances than all other cell populations investigated concerning their morphological adaptation (Supplementary Figure S4).

A quantification and length determination of the FAs (focal adhesions) was only possible on the controls and small spikes, since hardly any FAs were detectable on the other structures. The FA length was divided into six categories (resolution limit 0.5 µm) and the values of the small spikes were standardized to those of the controls. It became visible that for both time points and all cell types, the percentage of small FAs (0.5–1 µm) on the small spikes was larger than on the control and the percentage of larger FAs (>1.5 µm) decreased significantly (Figure 2H,I). The MC3T3-E1 had the highest percentage of small FAs with 334% after 24 h and 321% after 72 h. For the other cell types, this percentage was around 200% after 24 h and between 150% and 200% after 72 h. The percentage of FAs in the range of 1–1.5 µm was similar to the control in all different cells. With increasing FA length, the percentage decreased to a similar extent in all cell types when compared to the control until it reached almost 0% (24 h) and 0–28% (72 h) for 3–3.5 µm FAs.

### 2.3. The Spike Distance Influences Cell Migration of Peri-Implant Tissue Cells

**Figure 4:**
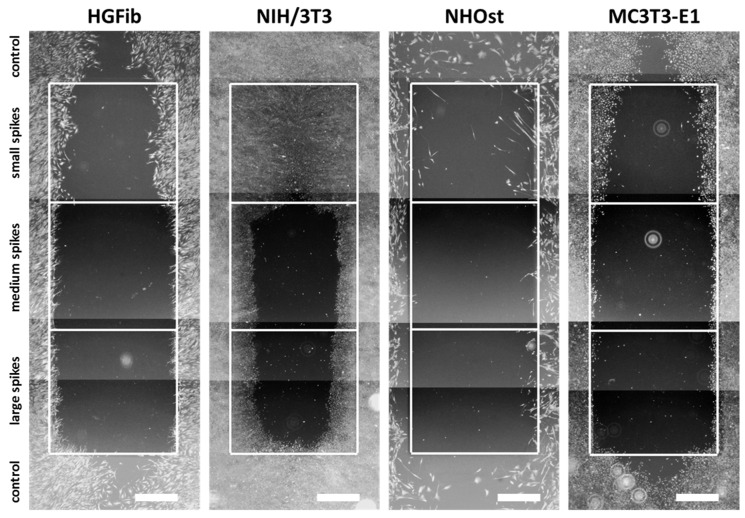
Cellular migration on spike topographies after 7 days. HGFib, NIH/3T3, NHOst, and MC3T3-E1 were cultivated for 7 days and stained with Calcein-AM. Scale = 1 mm.

**Figure 5:**
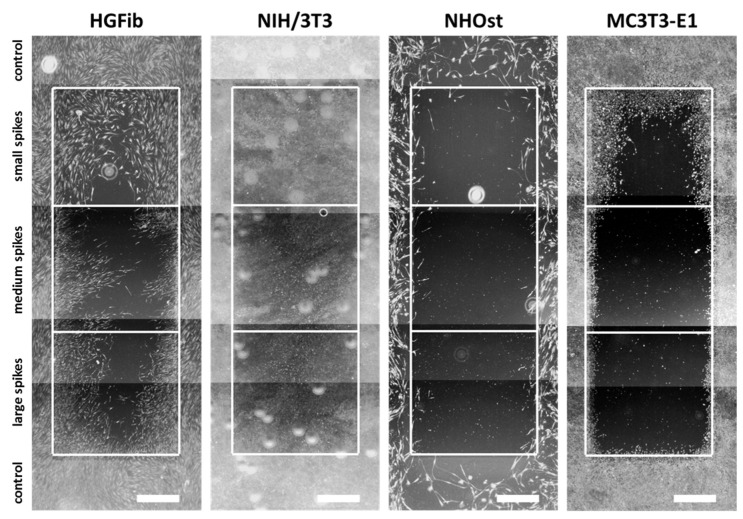
Cellular migration on spike topographies after 14 days. HGFib, NIH/3T3, NHOst, and MC3T3-E1 were cultivated for 14 days and stained with Calcein-AM. Scale = 1 mm.

The representative images illustrate the different migration behavior of different cells on structured substrates over time (Figure 4 and Figure 5 and Supplementary Figures S5–S7). After 3 days, the HGFib did not show any colonization of the structures, whereas the NIH/3T3 cells possessed a clear migration into the small spikes with an almost confluent cell front (Supplementary Figure S6). Furthermore, on the medium and large spikes, initial colonization was observed for the NIH/3T3. NHOst already showed sporadic colonization on the control surfaces, whereby only isolated cells penetrated mainly on the small spikes. For the MC3T3-E1, only a slight migration into the small spikes was observed (Supplementary Figure S6). After 7 days, a clear migration was observed for all cell types (Figure 4). This was most prominent for the NIH/3T3, which had completely colonized the small spikes. In addition, NIH/3T3 proceeded clearly on the medium and large spikes. HGFib and MC3T3-E1 migrated only into the small spike structures, while medium and large spikes were hardly colonized (Figure 4). NHOst continued their sporadic colonization of the structured areas (Figure 4). After 14 days, all cells on control surfaces reached confluency, except for NHOst. NIH/3T3 achieved complete colonization of all structured surfaces. HGFib migrated on all surfaces significantly, while the majority of colonization of both osteoblasts was limited to the small spikes (Figure 5).

**Figure 6:**
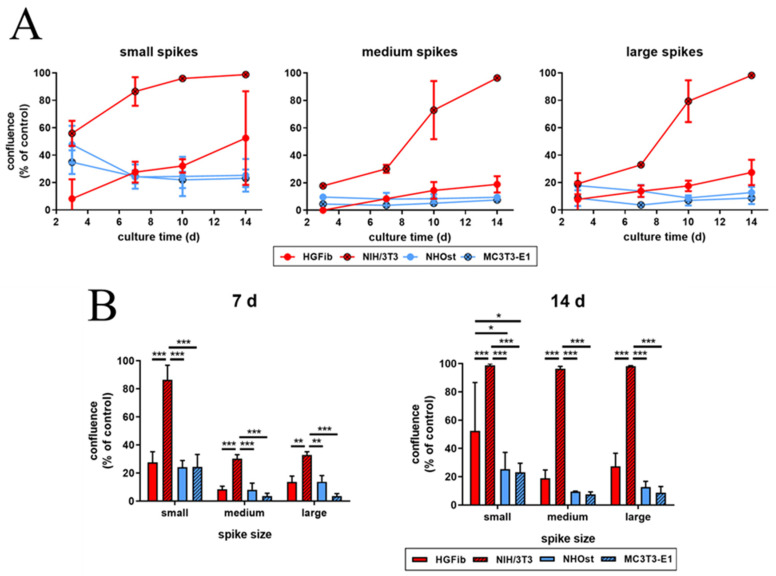
Cell migration of HGFib, NIH/3T3, NHOst, and MC3T3-E1 on spike topographies (direct comparison of the different cell type performances; for direct comparison of the different spike size effects, see Supplementary Figure S8). Cells were cultivated on the spike structures for 3, 7, 10, and 14 days and stained with Calcein-AM. The overgrown area was measured and normalized to each control. The course over an observation period of 2 weeks (**A**) and significant differences on day 7 and 14 (**B**) are presented. The mean values and standard deviation of three experiments are shown. Statistics were done by two-way-ANOVA (* *p* < 0.05, ** *p* < 0.01, *** *p* < 0.001).

The influence of spike distance on the migration of different cell types was confirmed after quantification (Figure 6 and Supplementary Figure S8). While every structure led to a significantly delayed migration in all cells, structures with a mean spike distance of 2.3 µm allowed intensive and fast cell growth throughout (Figure 6A). Already an increase in spike distance to 5.5 µm had a significant negative impact on cell migration, which was observed after one week (Figure 6B). However, this phenomenon did not increase further with large spike distances; instead, it seemed to be slightly higher (Figure 6B). It is noticeable that fibroblasts, after initial reduced migration on the structured surfaces, accelerated colonization over time compared to the control. The NIH/3T3 fibroblasts even reached confluence on all structures. In contrast, osteoblasts showed a smaller if not steadily slowing progression into the structured areas throughout the entire study period until confluence was reached on the control surfaces after 14 days for MC3T3-E1 (Figure 6B).

**Figure 7:**
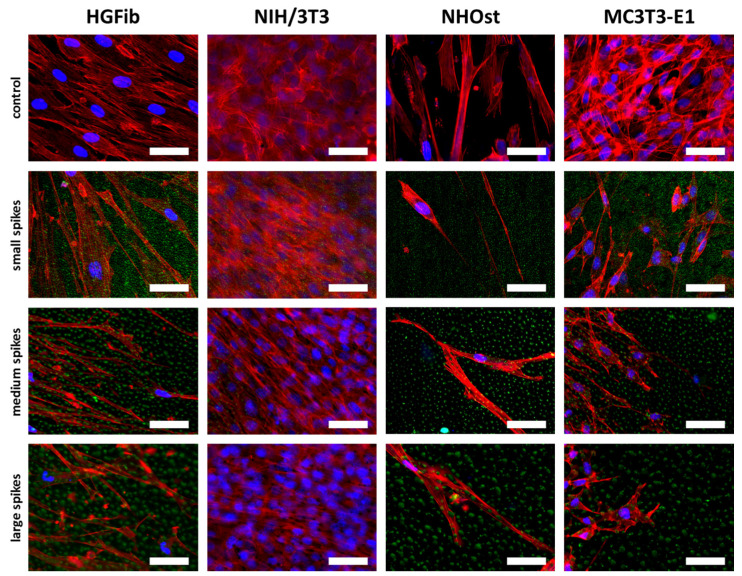
Cell morphologies of HGFib, NIH/3T3, NHOst, and MC3T3-E1 after migration on spike topographies. Cells on the spike structures (green) were fixed after 14 days and stained with DAPI (blue = nuclei) and phalloidin-TRITC (red = actin filaments). Scale = 50 µm.

Fibroblasts were orderly orientated on all structured surfaces during their migration, whereas their cell shape changed from fibroblastoid to elongated, while the size of the spike structure increased (Figure 7). However, this effect was more extreme for the HGFib, where the size and shape of the cell nucleus appeared influenced in addition to cytoskeletal irregularities. These effects were not observed to the same extent in osteoblasts. Changes in size and shape as well as morphological adaptation were less pronounced in osteoblasts, although the osteoblasts also showed a certain orientation on the structure (Figure 7).

## 3. Discussion

In the scientific field of biomedical research, a plethora of innovative materials, surface coatings, and structures are continuously developed every year to improve implant characteristics [17,18]. Despite the promising results of many studies, only a fraction reaches clinical implementation, as reduced functionality or biocompatibility often becomes apparent only in late stages of approval testing or even during early clinical trials [18]. So far, early-stage biocompatibility tests still focus on the cytotoxicity of a surface, which is required during approval process, as well as on the adhesion and the proliferation of cells on innovative implant material [41]. Moreover, immortalized and/or xenogenic cell lines, which slightly reflect the expected behavior of healthy human cells of the peri-implant tissue, have been used. Other important aspects of cell behavior for a successful tissue integration of implant material, such as cell migration or quality of adhesion, are not the focus of early screening schemes [23,28]. To avoid time-consuming and expensive in vivo investigations of ultimately deficient materials, a more reliable in vitro examination is required in order to detect any biocompatibility issues during the initial in vitro screening. In this way, time and costs would be saved and modifications to improve material biocompatibility would be addressed much earlier.

The SEM has been the standard method to investigate the interaction of tissue cells with steep microtopographies [42,43,44,45], although the reflection of laser signals during confocal microscopy has already been used to visualize fluorescent cells on topographies [46,47,48]. Furthermore, most cell culture assays used are terminal and are performed up to a few days [11]. Using CLSM instead of SEM enabled us to obtain faster quantification of the number, morphology, and detailed interaction of adherent cells with the peri-implant structure. Moreover, in combination with targeted optimizations of the cell culture assays, we could achieve a more detailed investigation of the material biocompatibility over a period of 14 days.

The screening approach we developed allows the detailed visualization of cell topography on and within implant surface structures as well as the investigation of adherence and proliferation. Thereby, it could be shown that the increase in spike dimension not only changes the size and shape of the cells, but also that the cells on medium and large spikes mainly reside between the structures or seemed to be perforated by them (Figure 3). The actin cytoskeleton was massively disturbed with parallel deformation of cell nuclei. The formation of strong actin bundles is an essential characteristic of vital cells, where the cell nucleus should be circular or oval due to the stabilization of the perinuclear actin cap [49,50]. A deformed nucleus can have a demonstrably negative effect on gene expression by shifting the lamina or chromosomes [42,51]. Below a certain value of cell area accompanied with a more elongated or rounded shape, proliferation no longer takes place, whereas extremely shrunk cells are often close to detachment from the substrate or even apoptosis [52]. Together with the significant shortening of focal adhesions (Figure 2H,I), which unfortunately could only be quantified on the small spikes due to the low resolution, these changes provide an explanation for the reduced adhesion and proliferation of cells, especially on spikes with a mean spacing of 5.5 µm and more (Figure 2A–C). Due to the new screening approach using CLSM and optimized cell culture assays, it could be demonstrated that the cell area, aspect ratio, actin cytoskeleton, nucleus, and length of focal adhesions were negatively affected by spike structures with a distance over 5.5 µm. Moreover, morphological changes could be correlated with altered cell adhesion and proliferation.

The frequent and partial detachment of cells is also an essential part of migration. In this case, cells are polarized and elongated, whereas the focal adhesions become less and shorter during cell motility [28,53,54]. Accordingly, additional migration assays may help to provide a comprehensive investigation of the cell adhesion phase on the structured surfaces. To provide a reliable and reproducible evaluation of cell migration, a cell exclusion assay was required, which allows the coverage of sensitive spike topographies before cell seeding and adhesion. Existing cell exclusion assays have been evaluated by light microscopy since the substrates were plastic materials, glass, or thin polymer layers [30,55,56,57,58,59,60]. To our knowledge, only Zhukova and colleagues have performed a cell exclusion assay on titanium so far [28]. Due to a special sputtering technique, they generated a 400 nm thin titanium layer and were able to use light microscopy for evaluation. Most new implant materials under investigation, including the spike structures used in this study, are solid, thicker, and non-transparent. Fluorescent dyes were therefore used to visualize the cells, but the labelling protocols had to be adapted to allow observation over long periods. During this study, Calcein-AM was repeatedly used and at a 40-fold higher dilution than recommended by the manufacturer to successfully prevent both bleaching and phototoxicity. Moreover, using an inverse microscope and glass bottom petri dishes, it was possible to work with the same cells under sterile conditions over 14 days. Regarding the migration of the different cell populations on the investigated topographies, it was observed that most massive restrictions emanated from the medium and large spike structures and were observed over the entire investigation period (Figure 6). Furthermore, the final microscopic images after 14 days confirmed the morphological and intracellular structural changes caused by the topographies during adhesion and proliferation of cells (Figure 7). The cell migration of different peri-implant cell types was successfully monitored over time and revealed their differences in migration behavior in relation to the spike distance.

Implemented as a simplified approach in initial screening processes, the quantification of focal adhesions allows a deeper evaluation of cell adhesion on implant materials and the effect of new structures [61,62]. The observation that the focal adhesions of all cell types grown on the small spikes were at the same level and the 3D models did not show any perforation of cells by the structures beneath suggests a flat orientation as on the smooth control (Figure 3). The focal adhesions were probably located on the tips of the spikes, as was the case in investigations of comparable microstructures [40,45]. On the medium and large spikes, the cells sank clearly into the structures (Figure 3). The resulting impairment of the cytoskeleton is an explanation for the absence of larger, detectable focal adhesions, since their formation and growth depend on the stress fibers and their force transmission [63,64]. A formation between the spikes used here is probably much more difficult than in comparable studies due to the depth of the structures and the superimposing nanotopography [40]. Nanostructures as well can influence cell attachment, whereby structures smaller than 70 nm or with a mean distance of more than 70 nm (which is the case in the presented structures) have a negative effect on the integrin grouping and thus, on the formation of focal adhesions [65]. A large cell area and fibroblastic morphology with numerous extensions on rigid surfaces is particularly characteristic of good cell adhesion [66,67] and goes hand in hand with well-developed focal adhesions, most of which are longer and more strongly coupled to the actin cytoskeleton in order to ensure optimal force transmission [28,49,50]. The combination of a nanotopography, which leads to the formation of small focal adhesions, and a microtopography, which causes a disturbance of the actin cytoskeleton, can thus explain the observed, particularly negative effects on adhesion and the processes dependent on it, such as proliferation and migration.

To further address the relevance of the used cell systems for the interpretation of cytocompatibility results regarding the intended functionality, frequently used immortalized murine cell lines (as a control) were compared to more relevant primary human cells. The differences as well as similarities between primary and immortalized fibroblasts or osteoblasts became prominent after quantitative analysis. According to the aspect ratio, after 72 h, the elongated shape of the primary cells was in strong contrast to the rounded shape of the immortalized cells (Figure 2F,G). The quantification of cell numbers, proliferation, and cell area demonstrated only a mismatch between HGFib and NIH/3T3, whereas NHOst and MC3T3-E1 performed similarly in these categories on all structures (Figure 2A–E). These results are supported by the observations of Czenska and colleagues, who were able to demonstrate several similarities between NHOst and MC3T3-E1 in vitro and concluded a possible use of immortalized cells as a substitute for primary cells in biomaterial testing, albeit not gene expression analysis [14]. The comparison of cell migration of primary and immortalized cells revealed in large parts the same tendency. On the surfaces, significant differences were just apparent between the fibroblasts, but not between the osteoblasts. Only NIH/3T3 were able to colonize all structures within 14 days, which was likely caused by the high pressure of a high proliferation rate on the unstructured surfaces as well as a multi-layered growth or a higher secretion of ECM proteins, which eventually support the migration on an otherwise unsuitable surface. However, a closer look at the behavior of the cells revealed that primary osteoblasts migrate differently as well, since HGFib, NIH/3T3, and MC3T3-E1 seem to migrate collectively and in close proximity to one another (Figure 4 and Figure 5). In contrast, it has been shown that single cells of the NHOst seemed to move fast by mesenchymal migration, whereas the majority of the cells remained sessile [50]. This indicates a heterogenic cell population, which is typical for primary cell isolates and therefore, better reflects the complex in vivo situation. In contrast, immortalized cells are arrested in one phase of differentiation and demonstrate a lower phenotypic variance [22]. Despite significant differences in FA length between MC3T3-E1 and the other cell types, this had no effect on cell proliferation or migration.

It is already known that cells from mice have a seven times higher metabolic rate than those from humans and tumor cells differ in size, proliferation, and metabolism from healthy counterparts, which in part might be an explanation for the described discrepancies [68,69]. However, due to a better availability and an easier cultivation of primary fibroblasts compared to primary osteoblasts, several studies compared primary fibroblasts with immortalized osteoblasts when analyzing the cell-specific effects of an implant surface [23,70,71]. As the results of this study illustrate, this approach can lead to false conclusions. The cell areas after 72 h as well as the proliferation activity of the HGFib on the small spikes were significantly increased compared to the MC3T3-E1, but not compared to the NHOst (Figure 2C,E). The proliferation and colonization activity of the immortalized MC3T3-E1 seemed to be impaired, which could be related to shorter FAs compared to the other cell types (Figure 2H,I). Further investigation is required in order to explain why small spikes affect the FA length especially in MC3T3-E1. Nevertheless, our investigations revealed important differences between primary and immortalized cells, which indicate that primary and immortalized variants of different cell types should not be compared in studies focusing on the cell-specific properties of implant modifications.

## 4. Material and Methods

### 4.1. Laser Structuring

Spike structures were created by ultra-short pulse laser ablation using the commercial femtosecond laser system “Femtopower Compact Pro” (Femtolasers Productions, Vienna, Austria) (parameters: pulse length: <30 s; central wave length: 796 nm; repetition rate: 1 kHz; maximum pulse energy: 800 µJ; beam diameter (1/e^2^): 15 mm; polarization: linear, horizontal). For structuring, samples of monocrystalline p-dotted silicium were placed in a process chamber with a pressure of 10 mTorr. The chamber was filled with SF_6_ (500 Torr) and placed on the X–Y-position system M-415.2S. Structuring was performed by moving the system line by line relative to the laser beam. Small spikes were produced with pulse energy of 5 µJ, medium spikes with pulse energy of 20 µJ, and large spikes with pulse energy of 30 µJ. The processed samples were sputtered with titanium to simulate a surface with comparable chemical composition to commercially available titanium implants [28] and finally, sterilized with 70% ethanol and 30 min UV exposure. Before each experiment, all samples were completely covered with PBS and air bubbles were thoroughly removed from the spike structures by pipetting to ensure a similar wettability. 

### 4.2. Cell Cultures

Primary human gingival fibroblasts (HGFib) were purchased from Provitro GmbH (Berlin, Germany). Immortalized murine fibroblasts (NIH/3T3), which are frequently used in studies for the investigation of surface topographies [44,45,72,73,74,75], were purchased from the Leibniz Institute (DSMZ GmbH, Braunschweig, Germany). Both fibroblast cell types were cultivated in DMEM (Biochrom, Berlin, Germany) supplemented with 10% FCS (PAN-Biotech, Aidenbach, Germany) and 1% *v/v* penicillin/streptomycin (Biochrom).

Primary human osteoblasts (NHOst) were purchased from Lonza (Basel, Switzerland) and cultivated in osteoblasts basal medium (Lonza) supplemented with 10% FCS (Lonza), 0.1% *v/v* ascorbic acid (Lonza), and 0.1% *v/v* gentamicin/amphotericin-B (Lonza). Immortalized murine osteoblasts (MC3T3-E1) are frequently used in studies for the investigation of surface topographies and implant surfaces [9,14,70,72,76,77,78] and were purchased from the American Type Culture Collection (Manassas, VA, USA). The cells were cultivated in αMEM (Lonza) supplemented with 10% FCS (PAN-Biotech) and 1% *v/v* penicillin/streptomycin (Biochrom).

Cell cultures were performed under the same conditions (37 °C, 5% CO_2_ in a humidified atmosphere) and cells were passaged at 80% confluence using trypsin/EDTA (5 min, 37 °C), blocked with the appropriate culture medium, and underwent subsequent centrifugation at 220–250× *g* for 5 min.

### 4.3. Immunofluorescence Staining for Quantification of Cell Adherence and Proliferation

In order to evaluate the cell adhesion and proliferation on spike-structured surfaces, cells were seeded on samples with 8000 cells/cm^2^ (in the case of HGFib and MC3T3-E1) or 5300 cells/cm^2^ (in the case of NHOst or NIH/3T3), respectively.

Cells were fixed after 24 or 72 h with cold 4% PFA (Carl Roth, Karlsruhe, Germany) in PBS for 20 min at 4 °C. In constant alternation, performed thorough washing steps with PBS, to block nonspecific binding, cells were first incubated with a solution of 2% BSA (Sigma-Aldrich, St. Louis, MO, USA) in PBS for 30 min at 37 °C before antibody staining of the focal adhesion complexes was performed—primary antibody: rabbit anti-pFAK (8556, Cell Signaling Technology, Danvers, MA, USA) overnight at 4 °C; secondary antibody: anti-rabbit DyLight488 (111-484-045, Jackson Immunoresearch Laboratories, Baltimore Pike, PA, USA) for 1 h at 37 °C; each was diluted 1:100 in 2% BSA, 0.3% Triton X^®^-100 (Sigma-Aldrich) in PBS and incubated in a humidified atmosphere. Finally, the cells were stained with phalloidin-TRITC 1:1000 and DAPI 1:10,000 in PBS for 15 min at room temperature.

**Figure 8:**
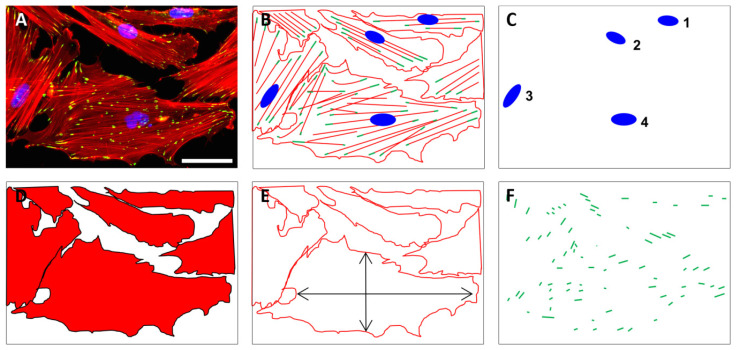
The quantification of cell numbers, cell area, aspect ratio, and focal adhesions on unstructured and structured surfaces was based on fluorescently stained cells. Exemplary fluorescence image of HGFib in the original (**A**) and in the schematic representation of stained cell components (**B**); the quantification of cell numbers was based on the DAPI-stained cell nuclei (blue) (**C**); the cell shape (**D**) as well as the aspect ratio of the cells (**E**) were derived from the staining of the actin fibers with phalloidin-TRITC (red); the pFAK antibody staining with DyLight488 (green) detection was used to determine the number, length, and composition of focal adhesion points (**F**). Scale = 50 µm.

Fluorescence microscopy (2D, Axio Scope A1, Zeiss, Goettingen, Germany) was applied using 10-fold magnification for the quantification of cell nuclei using DAPI staining as well as the cell area and aspect ratio using phalloidin staining. The quantification of pFAK staining was performed with images acquired at 40-fold magnification (Figure 8).

Confocal laser scanning microscopy (CLSM; SP8, Leica, Wetzlar, Germany) was applied using 63-fold magnification to visualize the cell morphology and spread on the different surface structures. DyLight488 and TRITC were excited at a wavelength of 488 nm, while the reflection of light at 638 nm was used to visualize the surface topography. Three-dimensional images were created by using the “surface mode” with the Imaris x64 6.2.1 software (Bitplane, Zurich, Switzerland).

The analysis of the nuclei, the cell area, and the cell shape was performed with ImageJ 1.46r (Wayne Rasband, National Institute of Health, Bethesda, MD, USA). For the quantification of the nuclei, the cell area, and the cell shape, stacks and binary images were created, intervening spaces were filled (“fill holes”), and nuclei or cells were separated (“watershed”). By synchronizing phalloidin–DAPI stacks with binary phalloidin stacks (“synchronize windows”), single cells could be identified and measured with the “wand tool”. For the quantification of the focal adhesions, the images were processed according to Horzum et al., 2014 [79]. The images were filtered with the “Log3D” function (settings: sigma X = 2, sigma Y = 2, slice per slice processing). Binary images were created by “default threshold” (settings: default, B&W, “set background pixels to NaN” was deactivated). The processed stack was synchronized with the RGB stack and the periphery of the cells was selected with “freehand selection”. The quantification was done by “analyze particles” (settings: size 0.1 cm^2^–infinity; circularity: 0–0.99, display results).

### 4.4. Migration Assay Optimized for Opaque Surfaces

**Figure 9:**
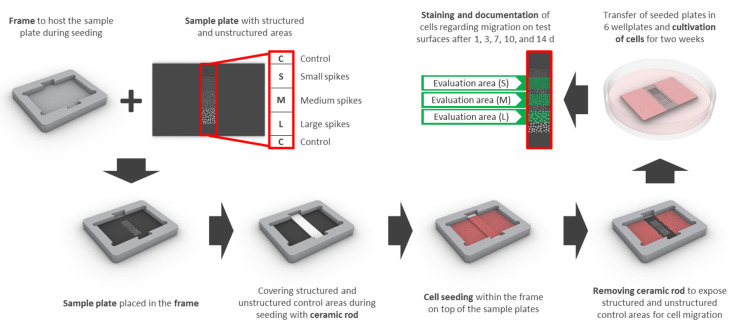
The design for the migration assay includes a sample plate with structured (small (S), medium (M), and large (L) spikes) as well as unstructured control areas (C) surrounding the structures and a frame, which serves as a placeholder for the sample plate and the ceramic rod. The areas of interest are covered with the ceramic rod before cell seeding and uncovered after initial adhesion to enable migration for 14 days. Quantification of stained cells was performed consecutively after 1, 3, 7, 10, and 14 days only in the designated structured areas (evaluation area (S), (M), or (L) in green) in order to avoid interference from adjacent areas.

In order to achieve a reproducible analysis of cell migration on non-transparent, structured surfaces, the following special devices and techniques were developed:

Samples had an edge length of 15 × 22 mm and contained 3 adjacent squares of different spike topographies (small, medium, large—see laser structuring), 3 × 3 mm each (S, M, L; Figure 9). Special frames were manufactured from titanium to host the samples and a partially covering ceramic rod for cell exclusion from the structured parts of the sample during cell seeding and initial adhesion (Figure 9). Frames were sterilized with 70% ethanol and washed with PBS prior to UV-sterilized samples being placed inside and transferred into 6 well plates. The ceramic rod (3.52 × 20 mm) was placed into the groove and 6 mL of a dense cell suspension was added (final seeding density: 18,750 cells/cm^2^ (HGFib), 12,500 cells/cm^2^ (NHOst), or 31,000 cells/cm^2^ (NIH/3T3 and MC3T3-E1), respectively). After 24 h, the ceramic rod was removed and the sample was transferred into an empty well. Cells were stained with 5 mL Calcein-AM (Thermo Fischer Scientific, Waltham, MA, USA) in PBS (25 ng/mL) at 37 °C for 30 min. Afterwards, samples were placed upside down in 35 mm petri dishes with a glass bottom (Ibidi, Planegg, Germany) filled with 3 mL DMEM without phenol red (Pan-Biotech, Aidenbach, Germany) including 10% FCS, 1% penicillin/streptomycin. Inverse fluorescence microscopy was performed at 490 nm excitation (520 nm emission) and indirect light was used for the visualization of the structure borders. After each capture, the samples were placed back into 6 well plates with fresh medium (original orientation) and cultivation was continued. The same procedure was repeated after 3, 7, 10, and 14 days.

For quantification, the proportion of structures overgrown with cells was measured using ImageJ (http://rsbweb.nih.gov/ij/), a public domain Java image processing program [80]. Only half of each structured area was considered for evaluation to reduce any influence of the non-structured adjacent areas. For comparability of the different cell types, the growth on the structured areas was normalized compared to that on the adjacent non-structured control surfaces (Figure 9).

### 4.5. Statistical Analysis

Significant differences were determined with GraphPad Prism 5.02 (GraphPad Software, La Jolla, CA, USA) using a two-way-ANOVA and subsequent Tukey test for multiple comparisons. * (*p* < 0.05), ** (*p* < 0.01), *** (*p* < 0.001).

## 5. Conclusions

In our study, we compared the attachment, growth, and migration behavior of primary and immortalized fibroblasts and osteoblasts on structured surfaces using extended analyses in the context of initial in vitro cytocompatibility screenings. In addition to the widely used quantification of adherent cells, we included a more detailed investigation of attachment quality by performing a three-dimensional reconstruction in confocal laser microscopy and by developing a new migration assay for long-term imaging optimized for opaque structured implant surfaces. Due to the illustrated difficulties in using immortalized cell lines to describe a possible tissue reaction or to influence cell-specific behavior based on topographies, we suggest the use of primary human cells in principle for future cytocompatibility assessments. If steep microtopographies are involved, the application of CLSM and the creation of 3D models can give additional information about the interaction of the cells with the surface topography. An analysis of cellular migration will further strengthen the quality of in vitro data during the initial cytocompatibility screening and support the process of clinical translation, since any biocompatibility issues and intended functionalities would be addressed much earlier.

## Supplementary Materials

Supplementary materials can be found at https://www.mdpi.com/1422-0067/21/22/8442/s1, Figure S1: Exemplary fluorescence images of HGFib, NIH/3T3, NHOst, and MC3T3-E1 after 24 h culture on flat control surfaces, small spikes, medium spikes and large spikes; Figure S2: Exemplary fluorescence images of HGFib, NIH/3T3, NHOst, and MC3T3-E1 after 72 h culture on flat control surfaces, small spikes, medium spikes and large spikes; Figure S3: Cell type-specific reactions on spike topographies (direct comparison of the different spike size effects); Figure S4: Exemplary 3D reconstructions of HGFib, NIH/3T3, NHOst, and MC3T3-E1 after 24 h culture on flat control surfaces, small spikes, medium spikes and large spikes; Figure S5: Cellular migration on spike topographies after 1 day. HGFib, NIH/3T3, NHOst and MC3T3-E1 were cultivated for 1 day and stained with Calcein-AM; Figure S6: Cellular migration on spike topographies after 3 days. HGFib, NIH/3T3, NHOst and MC3T3-E1 were cultivated for 3 days and stained with Calcein-AM; Figure S7: Cellular migration on spike topographies after 10 days. HGFib, NIH/3T3, NHOst and MC3T3-E1 were cultivated for 10 days and stained with Calcein-AM; Figure S8: Cell migration of HGFib, NIH/3T3, NHOst, MC3T3-E1 on spike topographies (direct comparison of the different spike size effects).
