# Supplementary material for: Cell Type-Specific Adhesion and Migration on Laser-Structured Opaque Surfaces
Source: Int J Mol Sci. 2020 Nov 10;21(22):8442. doi: 10.3390/ijms21228442 (PMC7696563; doi:10.3390/ijms21228442)
Supplement: Supplementary file 1 [file ijms-21-08442-s001.zip › ijms-935072-supplementary.docx]

**Supplementary Figures**


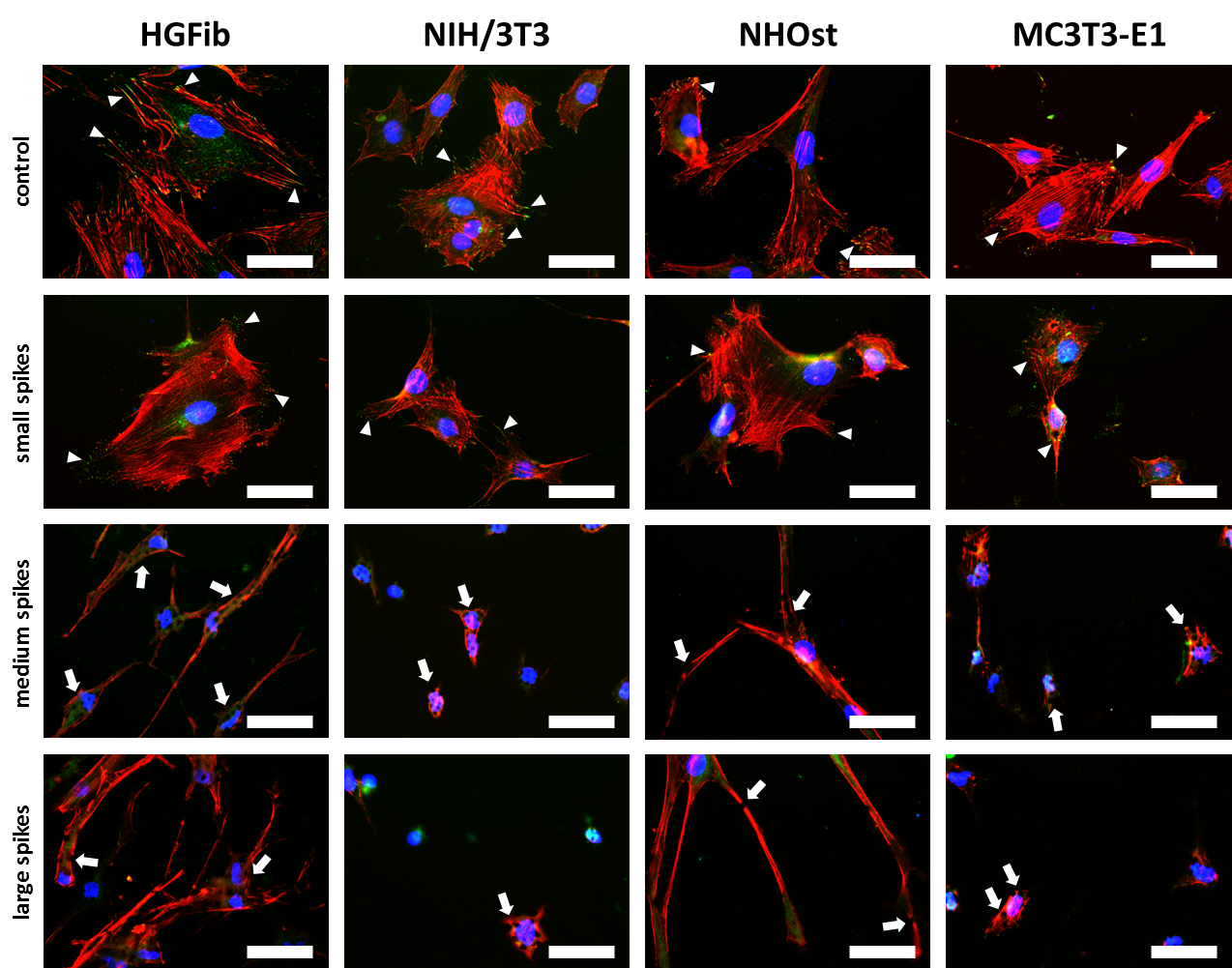


Supplementary Figure S1: Exemplary fluorescence images of HGFib, NIH/3T3, NHOst, and MC3T3-E1 after 24 h culture on flat control surfaces, small spikes, medium spikes and large spikes. Nuclei are stained with DAPI (blue), actin filaments with Phallodin-TRITC (red) and focal adhesions with DyLight488 (green). Highlighted are some focal adhesions (white arrowheads in pictures from control and small spikes) and holes in the cytoskeleton (white arrows in pictures from medium and large spikes). Scale = 50 µm.


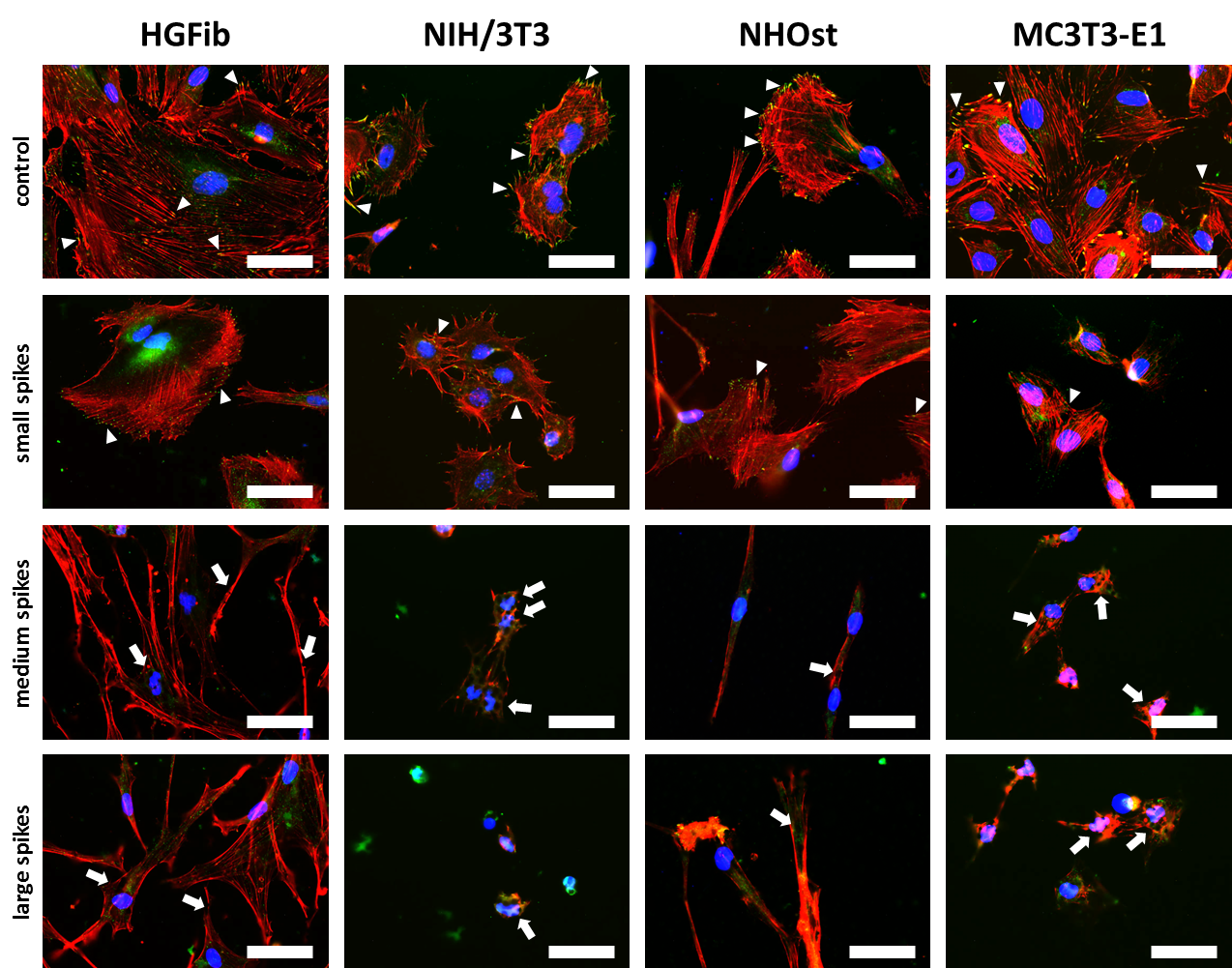


Supplementary Figure S2: Exemplary fluorescence images of HGFib, NIH/3T3, NHOst, and MC3T3-E1 after 72 h culture on flat control surfaces, small spikes, medium spikes and large spikes. Nuclei are stained with DAPI (blue), actin filaments with Phallodin-TRITC (red) and focal adhesions with DyLight488 (green). Highlighted are some focal adhesions (white arrowheads in pictures from control and small spikes) and holes in the cytoskeleton (white arrows in pictures from medium and large spikes). Scale = 50 µm.


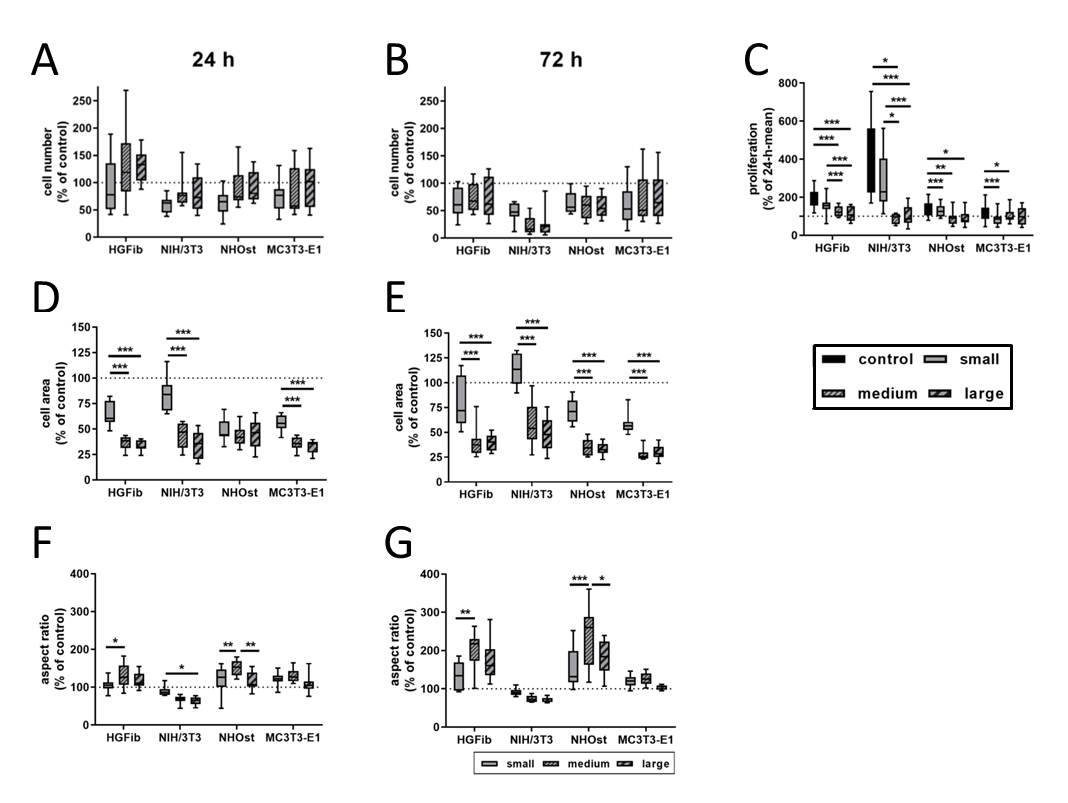


Supplementary Figure S3: Cell type-specific reactions on spike topographies (direct comparison of the different spike size effects; for direct comparison of the different cell type performances see Figure 2). HGFib, NIH/3T3, NHOst and MC3T3-E1 were cultivated for 24 h and 72 h on the spike structures. The cell number was determined by DAPI staining (A, B); the cell area (D, E) and aspect ratio (F, G) were determined by Phalloidin-TRITC staining. For calculation of cell proliferation cell numbers after 72 h were normalized to the corresponding 24 h mean value (C). Data is visualized by Box-Plots of 9 replicates of at least 3 independent experiments. Statistics were performed by two-way-ANOVA (*p < 0.05, **p < 0.01, ***p < 0.001).


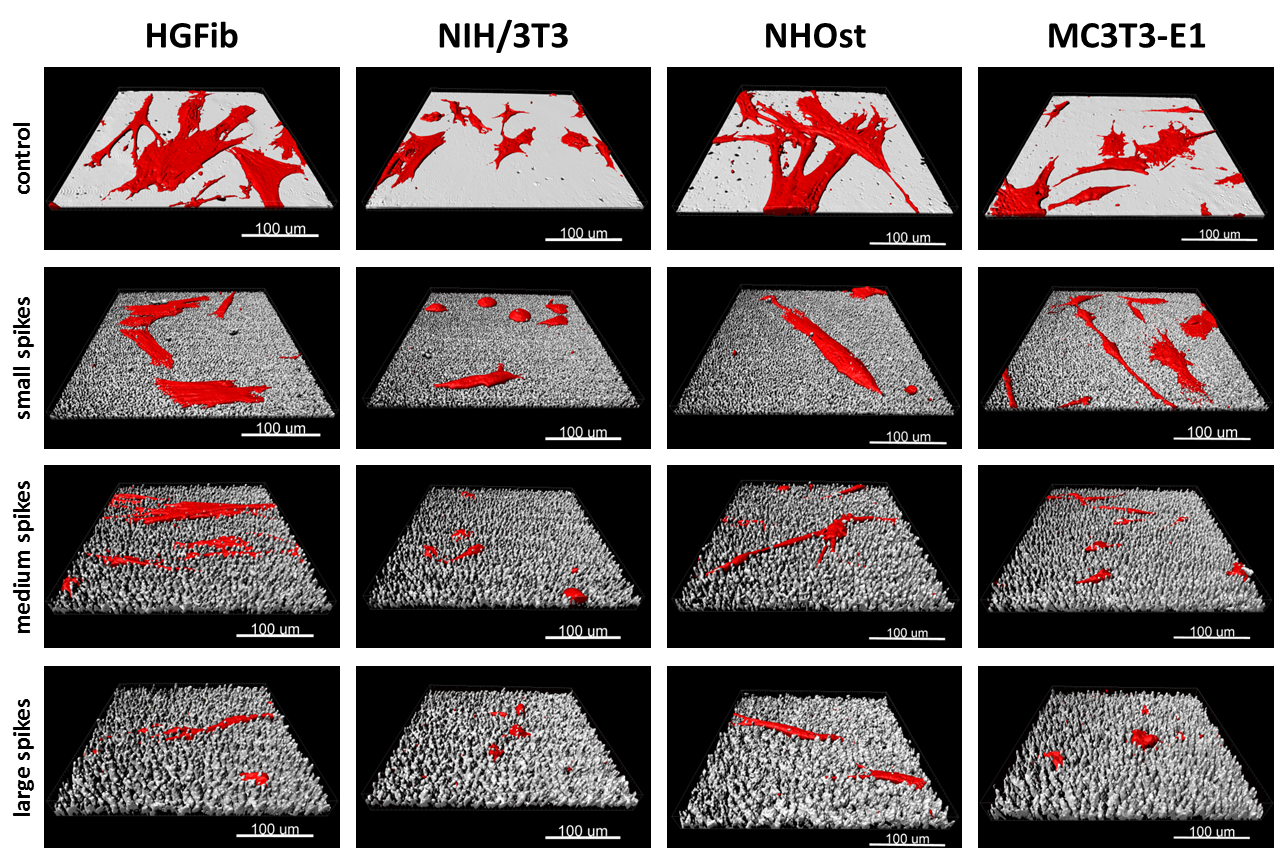


Supplementary Figure S4: Exemplary 3D reconstructions of HGFib, NIH/3T3, NHOst, and MC3T3-E1 (based on the actin filament staining with Phalloidin-TRITC) after 24 h culture on flat control surfaces, small spikes, medium spikes and large spikes; reflection of light at 638 nm was used to visualize the surface topography.


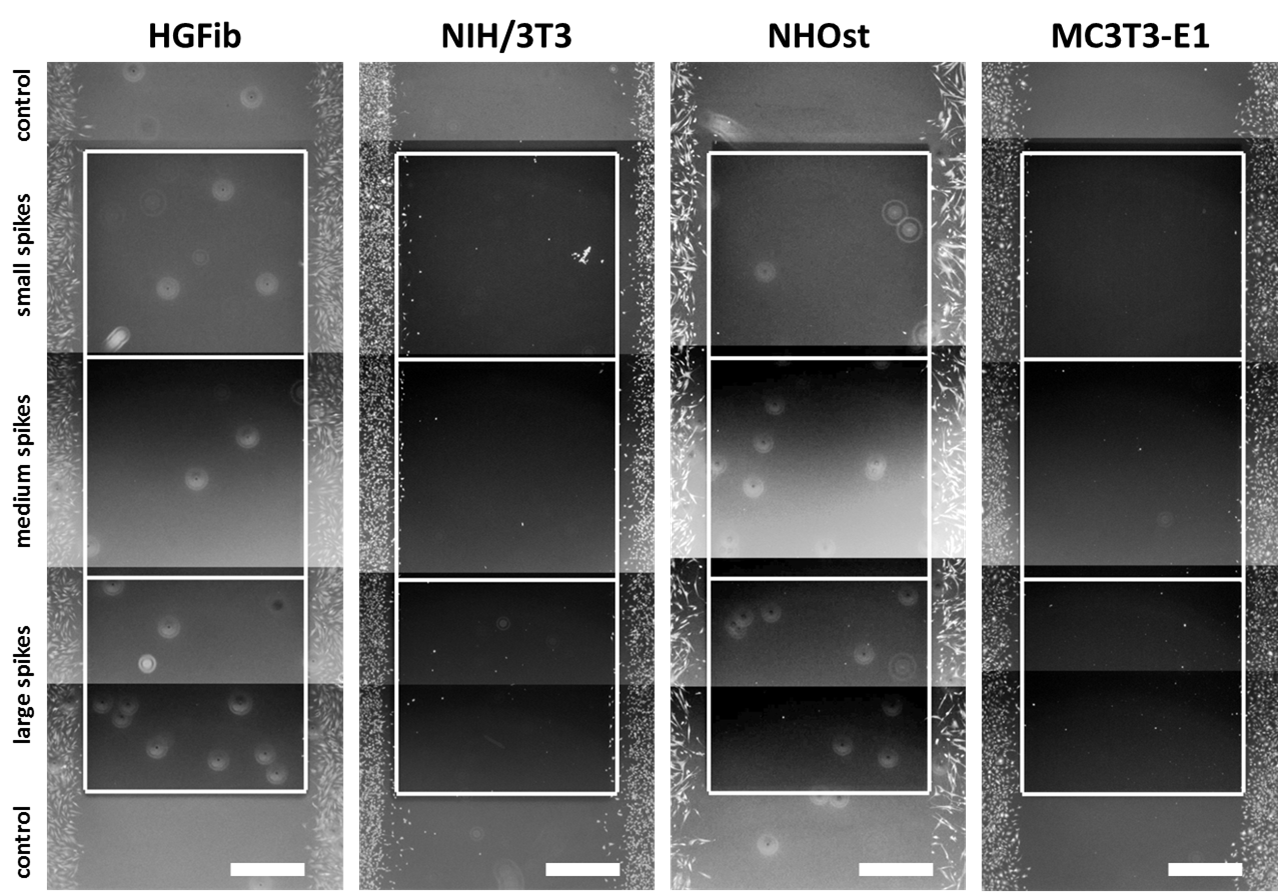


Supplementary Figure S5: Cellular migration on spike topographies after 1 d. HGFib, NIH/3T3, NHOst and MC3T3-E1 were cultivated for 1 d and stained with Calcein-AM. Scale = 1 mm.


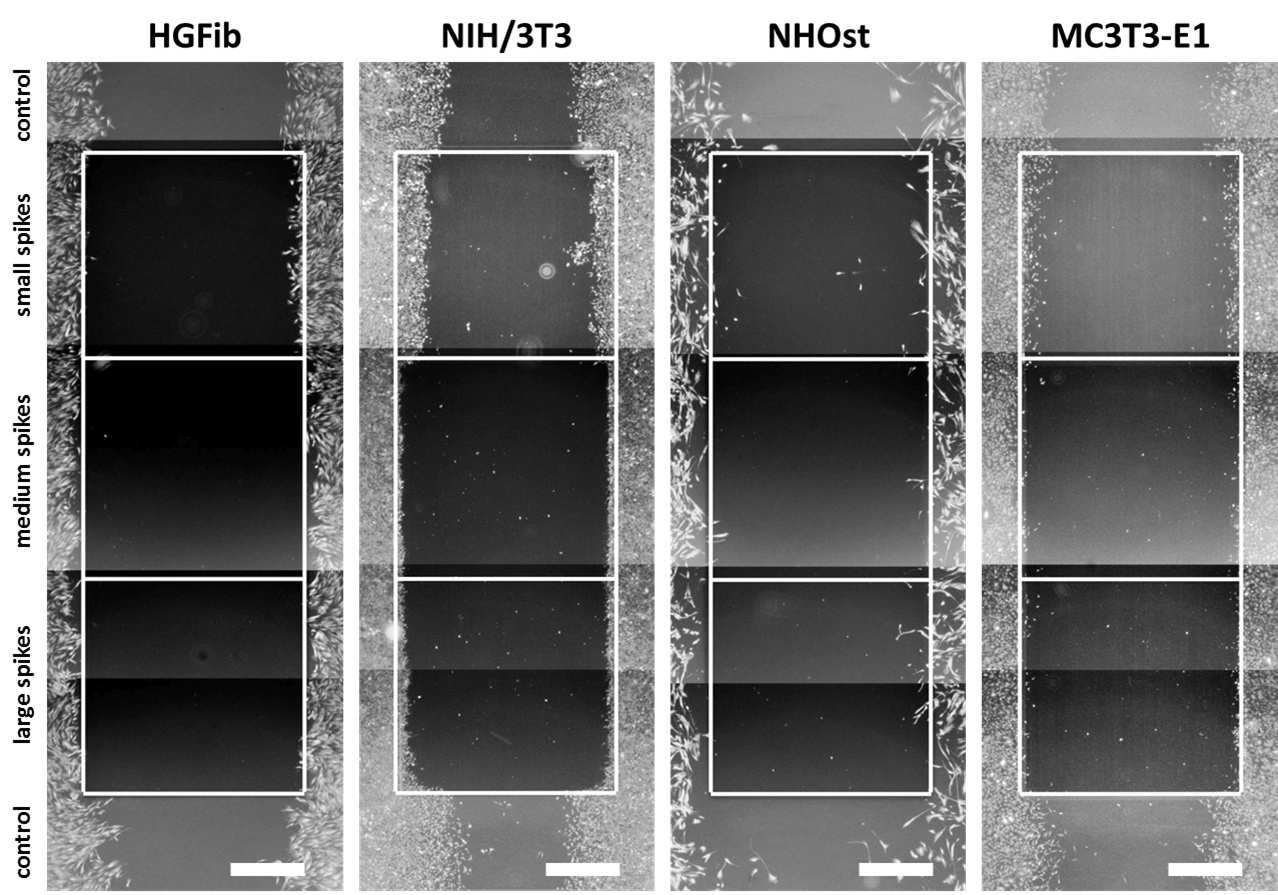


Supplementary Figure S6: Cellular migration on spike topographies after 3 d. HGFib, NIH/3T3, NHOst and MC3T3-E1 were cultivated for 3 d and stained with Calcein-AM. Scale = 1 mm.


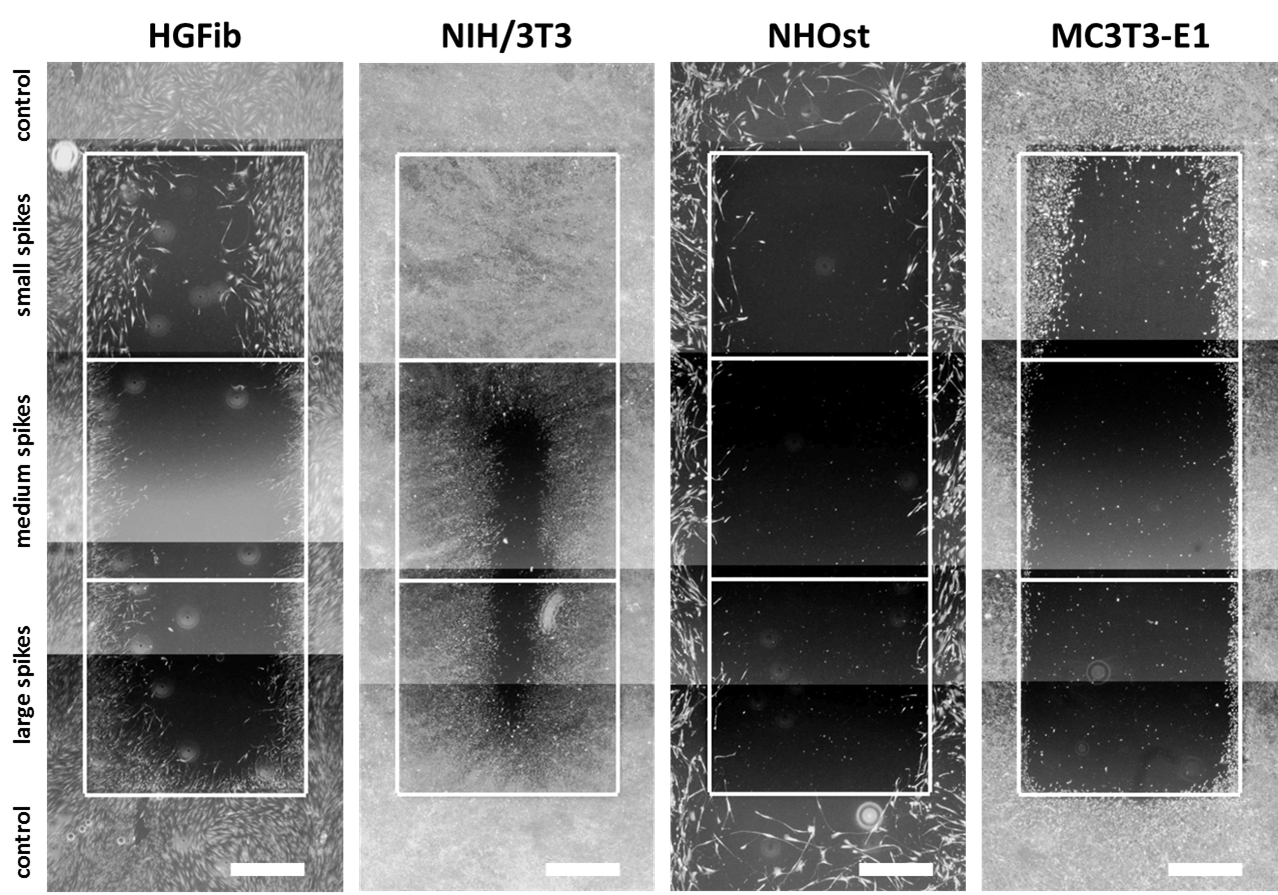


Supplementary Figure S7: Cellular migration on spike topographies after 10 d. HGFib, NIH/3T3, NHOst and MC3T3-E1 were cultivated for 10 d and stained with Calcein-AM. Scale = 1 mm.


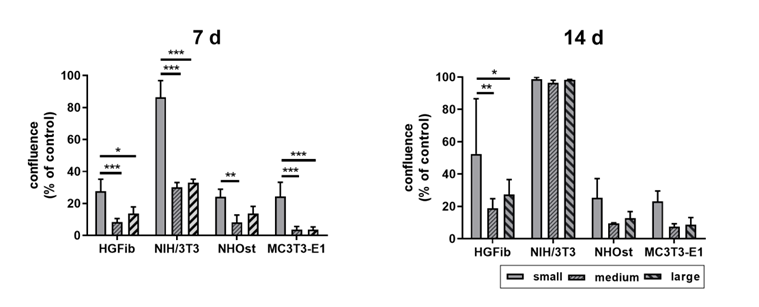


Supplementary Figure S8: Cell migration of HGFib, NIH/3T3, NHOst, MC3T3-E1 on spike topographies (direct comparison of the different spike size effects; for direct comparison of the different cell type performances see Figure 6). Cells were cultivated on the spike structures for 3, 7, 10 and 14 days and stained with Calcein-AM. The overgrown area was measured and normalized to each control. Significant differences on day 7 and 14 are shown. The mean values and standard deviation of 3 independent experiments are shown. Statistics were performed by two-way-ANOVA (*p < 0.05, **p < 0.01, ***p < 0.001).
